# Errors in agricultural practices increase the toxicity of heavy metals in the food chain at Ishwardi Upazila in Bangladesh

**DOI:** 10.1016/j.heliyon.2024.e29314

**Published:** 2024-04-05

**Authors:** Mahfuza Khanom Sheema, Zubaer Hosen, Afia Ibnath Shimki, Maisha Farzana Mim, Md Redwanur Rahman

**Affiliations:** a Institute of Environmental Science, University of Rajshahi, Rajshahi, Bangladesh; b Global Centre for Environmental Remediation, College of Engineering, Science and Environment, The University of Newcastle, Callaghan Campus, NSW, Australia; c Department of Pharmacy, Islamic University, Kushtia, Bangladesh; d Department of Food and Nutritional Science, Henry Institute of Bioscience and Technology, University of Rajshahi, Bangladesh

**Keywords:** Heavy metals, Contamination, Vegetables, Heath risk

## Abstract

Due to the potential harm to human health, heavy metal deposition in agricultural products has

gained importance throughout the world. Excessive use of agrochemicals and poultry wastes dramatically increased during the cultivation processes of rapidly growing vegetables without maintaining authorized guidelines. It happens due to the availability and low cost of these materials and higher production of the vegetables. Higher levels of heavy metals, especially Arsenic (As), Lead (Pb), Chromium (Cr), and Cadmium (Cd) contamination in food have detrimental effects on human health as well as the environmental ecosystems. This study revealed the profile of the heavy metals in the fast-growing vegetable called red amaranth (*Amaranthus cruentus*) due to the frequent use of poultry manure. We collected a total of 75 samples of red amaranth, water, and soil from five villages of five different unions at Ishwardi upazila in Bangladesh, and we analyzed the contamination levels of As, Pb, Cr, and Cd in them. Except for the As, we found that the accumulation levels of Pb, Cr, and Cd in samples crossed the highest permissive limit compared to FAO/WHO standards. Results suggested that daily intake of red amaranth in this area is alarming to human health due to the detrimental effects of these heavy metals.

## 1 Introduction

Due to the potential harm to human health, heavy metal deposition in agricultural products has gained importance worldwide. Metals such as arsenic (As), lead (Pb), chromium (Cr), and cadmium (Cd) are well-known carcinogens and have been reported to be exceptionally toxic even at very low concentrations [[1], [2], [3]]. They can collect in many body organs, are persistent in the environment, and are not biodegradable [4]. Contaminated soil through the use of long-term wastewater, excessive use of agrochemicals and poultry waste can result in heavy metal translocation into the soil, vegetables as well as food chain (90 % of total heavy metal contamination), and prolonged consumption of contaminated vegetables can pose a severe risk to the human body [[5], [6], [7], [8], [9], [10], [11], [12], [13], [14], [15], [16]] that leading to an increased incidence of chronic diseases such as cancer, deformity, endocrine disruptions, cardiovascular, nervous, kidney and bone diseases [[17], [18], [19], [20], [21], [22]]. Additionally, consumption of heavy metal-contaminated food can seriously deplete some essential nutrients in the body, causing a decrease in immunological defences, intrauterine growth retardation, impaired psycho-social behaviors, disabilities associated with malnutrition, and a high prevalence of upper gastrointestinal cancer [[23], [24], [25]].

Heavy metal-enriched ecosystem components result from rapid industrial development, improvements in agricultural chemicalization, and/or human activities in metropolitan areas [24,[26], [27], [28]]. Agricultural activities, especially the utilization of organic and inorganic fertilizer, the use of large quantities of pesticides, and the use of contaminated water, are the significant sources of trace metals caused by anthropogenic activities [29,30].

The primary anthropogenic sources of trace metals are agricultural operations, specifically using significant amounts of organic and inorganic fertilizers, pesticides, and contaminated water [2,31]. The roots and foilage were the main entry points for metals into the vegetable tissues, with root uptake being the predominant route. Research, however, revealed that leafy vegetables, followed by tubers and fruit vegetables, have the highest levels of metal enrichment in vegetables [32,33]. Therefore, the present study has been designed to assess and investigate the heavy metals (As, Pb, Cr, and Cd) contaminations in farmer's field soils, irrigated water, and most consumed leafy vegetables named red amaranth (*Amaranthus cruentus*) produced at Ishwardi Upazila of Pabna district, Bangladesh.

## 2 Materials and methods

### 2.1 Study areas

**Fig. 1:**
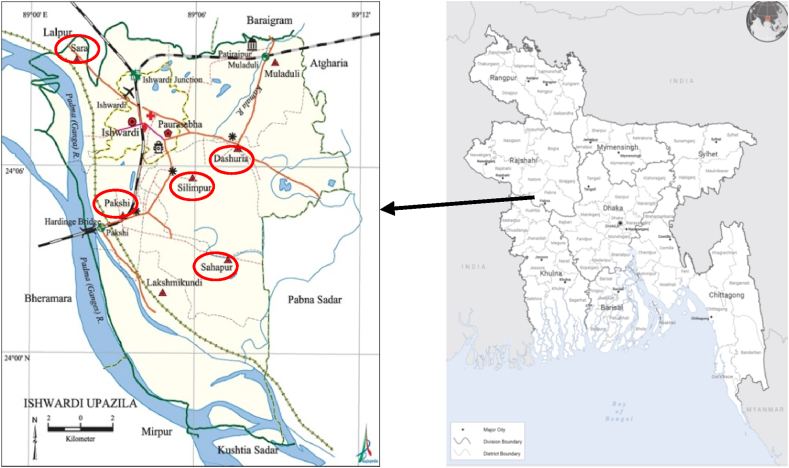
Magnified Ishwardi Upozella map from Bangladesh. The red circle represents the five unions from where we collected our specimens. (For interpretation of the references to colour in this figure legend, the reader is referred to the Web version of this article.)

The locations included private household vegetable gardens and commercial vegetable farms because they used the same techniques during crop production. Monitoring of environmental elements, including vegetables and soil in the Ishwardi area, was conducted to determine and measure human habituation. The results will provide crucial information for health-related objectives. The specimens, such as soil, water, and red amaranth, were collected from five sites of different unions across Ishwardi, Bangladesh, as shown in Fig. 1. Bohorpur village of Dasuria union, Sahapur village of Sahapur union, Ruppur village of Pakshi union, Mazadia village of Sara union, Boroichara village of Silimpur union. Besides vegetable farming, poultry farming is also the most popular in these areas, and poultry waste is used as organic fertilizer to reduce production costs. We selected a vegetable sample, red amaranth (*Amaranthus* *cruentus*) for detecting heavy metal contamination, which is the most consumed, has a low production cost and fast growth. We have collected 75 samples (15 per village) from our study sites.

### 2.2 Sample preparation

#### 2.2.1 Water collection and sampling

A total of 75 water samples (15 per village) were collected for the analysis. Samples were collected randomly from three different sources within the investigating area, including ponds, tube wells, and irrigation sources. Unfiltered water samples were collected using a falcon tube, placed into an icebox, and then stored in the refrigerator for analysis.

#### 2.2.2 Soil and vegetable collection and sampling

Following the previous report, we collected soil and red amaranth from our target sites [34]. Like water, we randomly collected 75 soil and vegetable specimens (15 per village). After placing the samples in little zipper bags, they were stored in the refrigerator for later analysis. All the red amaranth (*Amaranthus cruentus*) samples were washed with double-distilled water to get rid of airborne contaminants. To lower the water content, the samples' edible portions were weighed and air-dried for a day. To remove all moisture, all the samples were subsequently oven-dried for 24 h in a hot air oven at 70–80 °C. The powdered dried samples were sieved through a muslin cloth after being ground in a pestle and mortar.

#### 2.2.3 The veggie samples' digestion

Three 0.5 g powdered samples of each source of irrigation for each vegetable were precisely weighed and put in crucibles with three replicants. Then, they were placed in a muffle furnace. Perchloric acid and nitric acid (1:4) solutions were used to break down the ash. After being allowed to cool, the samples' contents were filtered using Whatman filter paper No. 42. With distilled water, each sample solution was diluted to a final volume of 25 ml before being subjected to atomic absorption spectrophotometry analysis.

#### 2.2.4 Instrumental analysis of heavy metals

Water, aqueous soil extracts, and red amaranth (*Amaranthus cruentus*) all contain high levels of heavy metals. In the Central Science Lab in Rajshahi, samples were analyzed using an atomic absorption spectrophotometer (AAS) with a GFA-7000A graphite furnace atomizer (SHIMADZU, AA- 7000, Japan). All processes were performed according to the report [35].

#### 2.2.5 Statistics

All data were analyzed using IBM SPSS software (version 29.0). Descriptive statistics were performed to calculate mean ± SD.

## 3 Results and discussions

The profile of heavy metal concentrations found in our research area's irrigation water, soil, and red amaranth (*Amaranthus cruentus*) is shown in Table 1. This table also compared the area-based contaminations. Results suggested that, in our areas, irrigation water is not liable for this contamination. Cr, Cd, and Pb concentrations in soil and vegetables were higher everywhere. A wide range of values for metal concentrations was observed among the sampling sites. Factors such as salinity, geomorphological setup, farming process, and land runoff might have played a crucial role in the variation of metals [36]. As shown in Table 1, concentrations of heavy metals at some sites were much higher than others because these sites are located downstream of the river, with extensive discharging of urban waste, excessive use of agrochemicals, and use of poultry litter (Composed of poultry feed, waste, and blending of rice husk) as organic fertilizer. Cr, Cd, and Pd concentration in sediment was higher than As because using tannery waste as feed in poultry farming and excessive use of agrochemicals, a common practice in most of Bangladesh's agroecological zones with high Cr, Cd, and Pb levels may be responsible for the heightened levels of these metals in the vegetables [12].

**Table 1:** Heavy metals profiling (concentration) in water, Soil, and vegetables in the study areas.

Metals	Areas														
	Bohorpur			Sahapur			Ruppur			Mazdia			Boroichara		
	Water*	Soil	V1	Water*	Soil	V2	Water*	Soil	V3	Water*	Soil	V4	Water*	Soil	V5
As (mg/kg)	0.003 ± 0.01	0.23 ± 0.07	0.17 ± 0.04	0.256 ± 0.23	0.38 ± 0.15	0.26 ± 0.02	0.052 ± 0.07	0.18 ± 0.05	0.15 ± 0.05	0.309 ± 0.21	0.33 ± 0.15	0.28 ± 0.10	0.006 ± 0.01	0.16 ± 0.04	0.11 ± 0.04
Pb (mg/kg)	0.072 ± 0.01	3.02 ± 0.48	2.33 ± 0.41	1.060 ± 0.65	2.77 ± 0.43	3.12 ± 1.38	0.064 ± 0.02	3.01 ± 0.70	2.19 ± 0.63	0.068 ± 0.01	3.21 ± 0.80	2.65 ± 0.77	1.072 ± 0.64	4.32 ± 0.97	3.97 ± 0.96
Cr (mg/kg)	2.184 ± 0.48	8.42 ± 0.89	7.17 ± 0.79	0.039 ± 0.04	20.7 ± 3.60	11.3 ± 1.74	1.986 ± 0.79	8.89 ± 1.20	9.22 ± 1.40	0.108 ± 0.05	10.71 ± 1.63	2.95 ± 1.14	0.061 ± 0.06	9.75 ± 1.83	4.67 ± 1.20
Cd (mg/kg)	0.014 ± 0.01	0.73 ± 0.11	0.24 ± 0.07	0.014 ± 0.01	0.32 ± 0.05	0.26 ± 0.06	0.013 ± 0.01	0.80 ± 0.13	0.19 ± 0.02	0.004 ± 0.01	0.54 ± 0.15	0.41 ± 0.15	0.005 ± 0.01	0.86 ± 0.26	0.28 ± 0.14

Notes: Data are presented as Mean ± SD. *Unit of water is mg/l; V, Vegetable; As, Arsenic; Pb, Lead; Cr, Chromium; Cd, Cadmium.

**Fig. 2:**
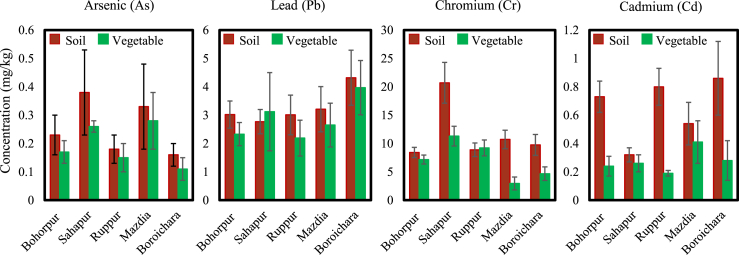
Heavy metal accumulation from soil to vegetables. Data were presented as Mean ± SD. The dark red bar indicates the heavy metal concentrations in soils, and the green bar indicates the heavy metal concentrations in vegetables. (For interpretation of the references to colour in this figure legend, the reader is referred to the Web version of this article.)

Heavy metal buildup in crops from the respective soils is depicted in Fig. 2. According to our investigation , the levels of contamination in vegetables were typically lower than those in equivalent soils. This may be explained by the root, which appears to act as a barrier to metal transfer. However, it has been noted that heavy metals from soil can accumulate in vegetables [37,38]. By calculating the transfer factor for the soil/plant system, it was possible to quantitatively assess the relationship between the concentration of metals in vegetables and the corresponding soils [[39], [40], [41]]. Most of the time, plants only absorb one or two types of heavy metals from the soil solution. The transfer of metals from soils to plants depends on a variety of elements, including metal forms, plant species and parts, and soil characteristics [42]. The pH and the redox potential of the soil or the root system significantly impact the solubility and, as a result, the plant uptake of trace metals in the soil [43]. The related anion and particle size significantly influence plant development and metal uptake [39,44]. If the concentration is within a particular range, plants tend to absorb more heavy metals as concentrations rise. As a result, plant roots are damaged, and when the concentration exceeds the range, there will be less absorption [45].

**Fig. 3:**
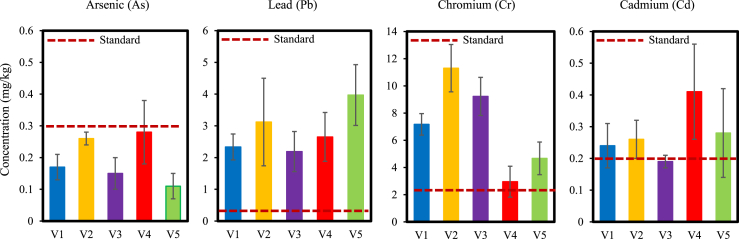
Comparison of heavy metal concentration in vegetables according to FAO/WHO standards (Highest permissible limit), As, 0.3 mg/kg; Pb, 0.3 mg/kg; Cr, 2.3 mg/kg and Cd, 0.2 mg/kg. Data were presented as Mean ± SD. Here collected vegetable samples V1, Bohorpur; V2, Shahapur; V3, Ruppur; V4, Mazdia; and V5, Boroichara. The dark red dashes marked the highest permissible limits of As, Pb, Cr, and Cd, respectively. (For interpretation of the references to colour in this figure legend, the reader is referred to the Web version of this article.)

By diluting the metal concentration in accordance with the ratio of FW and DW, metal concentrations determined on a dry weight basis (DW) were changed to a fresh weight basis (FW). According to FAO/WHO guidelines, Fig. 3 compares the levels of heavy metal contamination in vegetables [46]. The graph shows that, compared to the reference value (0.3 mg/kg), As concentrations are lower in all locations. The other metals such as Pb, Cr, and Cd pollution is higher than the recommended levels. According to Ref. [47], the primary way that heavy metal contamination affects humans is through their food chain. In red amaranth, a staple vegetable in those regions, we discovered a significant concentration of Cr, Cd, and Pb (Fig. 3). It is well known that elevated levels of Cr, Cd, and Pb have several toxic effects on the body, including hypertension, kidney malfunction, damage to lungs, hepatic, skeletal, and reproductive malformations, allergic reactions in the skin, triggering of asthma attacks and long-term exposure of the metals is liable to cellular damage, mutation of cells and even cancer [48,49]. We only analyzed the levels of heavy metal concentrations (As, Pb, Cr, and Cd) in the collected red amaranth. However, we did not go for the health assessment because of the funding and time limitations. This may be a possible ground for future research.

## 4 Conclusions

The current study found that Cr, Cd, and Pb concentrations were higher than the recommended values, indicating that anthropogenic activities have contaminated the riverside area of Padma with heavy metals, which could hurt both human health and the riverine ecosystem. Three of four heavy metal accumulations in red amaranth crossed the FAO/WHO recommended maximum permissive limits in most areas. Long-term and daily intake of the contaminated vegetable has detrimental effects even after low doses. To prevent an excessive build-up of these hazardous heavy metals from effluents and sewage in vegetables and other food materials, constant monitoring is necessary.

## Funding

This research did not receive any specific grant from public, commercial, or not-for-profit funding agencies.

## Data availability

Data will be made available on request.

## CRediT authorship contribution statement

**Mahfuza Khanom Sheema:** Writing – original draft, Data curation. **Zubaer Hosen:** Writing – review & editing, Validation, Formal analysis. **Afia Ibnath Shimki:** Writing – original draft, Data curation, Conceptualization. **Maisha Farzana Mim:** Writing – original draft, Data curation, Conceptualization. **Md Redwanur Rahman:** Writing – review & editing, Supervision, Methodology.

## Declaration of Competing interest

The authors declare that they have no known competing financial interests or personal relationships that could have appeared to influence the work reported in this paper.
